# First identification of an ST11-KL64 hypervirulent *Klebsiella pneumoniae* strain coproducing KPC-2 and NDM-1 with an *OmpK36* GD mutation

**DOI:** 10.3389/fmicb.2026.1755521

**Published:** 2026-02-16

**Authors:** Jiaoli Chen, Cheng Wang, Lingbin Wu, Ziling Lan, Mei Li, Jianfen Xu, Xiaolei Hu, Jiansheng Huang

**Affiliations:** 1 Department of Clinical Laboratory, The Fifth Affiliated Hospital of Wenzhou Medical University, Lishui, Zhejiang, China; 2 Department of Clinical Laboratory, Lishui Second People's Hospital Affiliated to Wenzhou Medical University, Lishui, Zhejiang, China; 3 Department of the School of Medical Technology and Information Engineering, Zhejiang Chinese Medical University, Hangzhou, Zhejiang, China

**Keywords:** hypervirulent *Klebsiella pneumoniae*, KPC-2, NDM-1, *OmpK36* GD mutation, ST11-KL64

## Abstract

**Introduction:**

The convergence of hypervirulence and carbapenem resistance in *Klebsiella* pneumoniae represents a critical clinical threat.

**Methods:**

We characterized 27 NDM-producing *K. pneumoniae* isolates collected from a tertiary hospital in Lishui, China, between 2017 and 2023. Among the isolates, we selected an ST11 strain, CRKP26, carrying *bla*_KPC-2_ and *bla*_NDM-1_, for further investigation using whole-genome sequencing (WGS) and phenotypic assays.

**Results:**

The surveillance detected high clonal diversity across 19 sequence types among the isolates. CRKP26 exhibited extensive drug resistance, with resistance to ceftazidime, cefepime, aztreonam, piperacillin-tazobactam, and amikacin and high-level carbapenem resistance, with MICs of 64 μg/mL for imipenem and 256 μg/mL for meropenem. This strain was susceptible only to polymyxin B and tigecycline. Whole-genome sequencing (WGS) of CRKP26 revealed that *bla*_KPC-2_ and *bla*_NDM-1_ were located on separate plasmids and that insertion of glycine–aspartate (GD) at positions 137–138 was identified in the *ompK36* gene. WGS further identified CRKP26 as capsular serotype KL64 and confirmed the presence of core virulence determinants, including the aerobactin operon (*iucABCD*-*iutA*), on an IncFIB virulence plasmid. Strain CRKP26 was defined as hypervirulent (LD_50_ ≤ 1 × 10^6^ CFU), despite showing slightly attenuated lethality compared to the classic hypervirulent strain NTUH-K2044. Furthermore, the survival rate of CRKP26 was 93.2% in serum killing assays and 90.7% in neutrophil killing assays, comparable to that of the hypervirulent control NTUH-K2044.

**Conclusion:**

To our knowledge, this is the first report of a hypervirulent ST11-KL64 *K. pneumoniae* strain carrying both *bla*_KPC-2_ and *bla*_NDM-1_ with an *OmpK36* GD mutation. This convergence of plasmid-mediated resistance, chromosomal mutation, and hypervirulence in a high-risk clone highlights an urgent threat requiring increased genomic surveillance.

## Introduction

*Klebsiella pneumoniae* is a major pathogen that causes severe nosocomial infections and readily acquires antimicrobial resistance (Karampatakis et al., 2023). Carbapenem-resistant *K. pneumoniae* (CRKP), listed as a critical priority pathogen by the World Health Organization, represents one of the most urgent global threats (Liang et al., 2022; Wu et al., 2022). *Klebsiella pneumoniae* carbapenemase (KPC) and New Delhi metallo-*β*-lactamase (NDM) are the two most prevalent carbapenemases worldwide and represent critical threats to antimicrobial therapy (Argimon et al., 2021; Feilong et al., 2025).

In China, CRKP accounts for 64% of carbapenem-resistant *Enterobacterales* infections, with ST11-*bla*_KPC-2_ representing the dominant epidemic lineage (Shi et al., 2024; Liu et al., 2022; Jing et al., 2022). More concerning is the emergence of strains coproducing both KPC and NDM. KPC, a highly efficient serine carbapenemase, confers pan-β-lactam resistance, whereas NDM, a metallo-β-lactamase, confers additional resistance to novel β-lactamase inhibitor combinations such as ceftazidime/avibactam (Li et al., 2024; Zhang et al., 2025). The coproduction of these complementary carbapenemases results in extensively resistant phenotypes and significantly increased mortality rates (Li et al., 2024; Wang et al., 2024). While most CRKP strains harbor a single carbapenemase gene, the proportion of KPC/NDM coproducers has increased in recent years (Guo et al., 2023; Vivas et al., 2020; Ceron et al., 2023; Vasquez-Ponce et al., 2022). This emergence has been documented globally, including in recent reports from Paraguay and Brazil, where ST11 remains a dominant background for the co-occurrence of *bla*_KPC-2_ and *bla*_NDM_ (Melgarejo Touchet et al., 2025; Berdichevski et al., 2025). Furthermore, mutations in outer membrane porins, particularly the *OmpK36* GD mutation, synergize with carbapenemases to further increase resistance. The *OmpK36* GD mutation causes porin constriction, reducing pore size by approximately 26% and restricting antibiotic penetration, thereby increasing carbapenem MICs in ST258/ST11 CRKP (Wong et al., 2019; David et al., 2022; Rogers et al., 2023). This combination of multiple resistance mechanisms—the production of two carbapenemases coupled with porin defects—represents a formidable therapeutic challenge.

In addition to increasing carbapenem resistance, the emergence of hypervirulent *K. pneumoniae* (hvKP) as a distinct pathogenic variant presents another clinical challenge. Unlike classic *K. pneumoniae* (cKP) strains, hvKP strains cause severe community-acquired infections in healthy individuals (Al Ismail et al., 2024; Hetta et al., 2025). This enhanced virulence is attributed to large virulence plasmids harboring genes for aerobactin (*iucABCD-iutA*), salmochelin (*iroB-N*), and capsule regulators (*rmpA/rmpA2*), which confer enhanced iron acquisition, immune evasion, and hypermucoviscous phenotypes. The convergence of carbapenem resistance and hypervirulence in *K. pneumoniae* represents a serious clinical challenge. In China, this convergence has been documented primarily in ST11 strains that acquired pLVPK-like virulence plasmids, resulting in high-mortality outbreaks (Zhou et al., 2020; Gu et al., 2018). ST11 hypervirulent strains coproducing both KPC and NDM have been recently identified and exhibit pan-*β*-lactam resistance and lead to high mortality rates (Wang et al., 2024; Han et al., 2022).

Previous research on carbapenem-resistant hypervirulent *K. pneumoniae* has emphasized the plasmid-mediated transfer of resistance and virulence genes (Wang et al., 2024; Liu et al., 2024). However, the combined contribution of dual-carbapenemase production and chromosomal porin mutations in hypervirulent strains remains poorly understood. As of the time of this study (October 2025), no report about the *Klebsiella pneumoniae* strain coproducing KPC-2 and NDM-1 with an *OmpK36* GD mutation has been found in public databases.

Here, we investigated the molecular epidemiology of 27 NDM-producing *K. pneumoniae* isolates from a tertiary hospital in Lishui, China, which exhibited high clonal diversity (19 distinct sequence types). Among these, we identified and comprehensively characterized CRKP26, a hypervirulent ST11-KL64 strain. Our analysis revealed that this strain uniquely combines the plasmid-mediated dual carbapenemase genes *bla*_KPC-2_ and *bla*_NDM-1_, a chromosomal *OmpK36* GD mutation, and plasmid-borne hypervirulence determinants (*iucABCD-iutA*), resulting in both high-level carbapenem resistance and increased pathogenicity.

## Materials and methods

### Carbapenem-resistant *K. pneumoniae* isolates collection

In total, 94 carbapenem-resistant *K. pneumoniae* isolates were collected from clinical specimens in a tertiary hospital in Lishui, China, between January 2017 and December 2023. The presence of the *bla*_NDM_ gene was confirmed by PCR and sequencing; only 27 *bla*_NDM_-positive strains were included in this study.

### Bacterial identification and antimicrobial susceptibility testing

Species identification was performed using VITEK MS. MICs were determined by the broth microdilution method according to CLSI 2024 standards; the results with tigecycline were interpreted according to FDA guidelines. The quality control strains included *E. coli* ATCC 25922, *P. aeruginosa* ATCC 27853, and *S. aureus* ATCC 25923.

### Detection of carbapenem resistance genes

PCR amplification was performed to detect a broad panel of resistance genes, including carbapenemases (*bla*_NDM_, *bla*_KPC_, *bla*_VIM_, *bla*_IMP_, *bla*_GES_, *bla*_SME_, *bla*_IMI_, *bla*_SIM_, *bla*_GIM_, *bla*_SPM_, *bla*_OXA-23_, *bla*_OXA-24_, *bla*_OXA-58_), extended-spectrum *β*-lactamases (*bla*_CTX-M_, *bla*_TEM_, *bla*_SHV_), and plasmid-mediated quinolone resistance genes (*qnrA, qnrB, qnrC, qnrD, qnrS, qepA, aac (6′)-Ib-cr*). Primers were designed based on the conserved regions of the target genes using Primer Premier 5.0 software. The specific primer sequences are listed in Supplementary Table S1. All primers were synthesized by Qingke Biotechnology Co., Ltd. (Hangzhou, Zhejiang Province, China). Products were analyzed by 1% agarose gel electrophoresis at 120 V for 30 min and subsequently sequenced. The sequences were compared against the NCBI BLAST database for confirmation.

### MLST

Multilocus sequence typing was performed by PCR amplification and sequencing of housekeeping genes using primers and conditions provided by the *K. pneumoniae* MLST database (https://bigsdb.pasteur.fr/klebsiella/). Allele profiles were assigned to sequence types.

### Plasmid typing

*Bla*_NDM_-carrying plasmids were transferred into Trans1-T1 competent cells or J53 cells via transformation or conjugation. Transformants/conjugants were confirmed by VITEK MS and PCR. Plasmids were extracted using an EasyPure MiniPrep Kit and typed by PCR targeting plasmid replicons.

### Whole-genome sequencing and gene context analysis

Genomic DNA from the *bla*_NDM_/*bla*_KPC_-positive ST11 strain CRKP26 was extracted using an EasyPure Bacteria Genomic DNA Kit. Whole-genome sequencing was performed using a hybrid strategy of combining next-generation sequencing (NGS) on an Illumina NovaSeq platform with third-generation single-molecule sequencing on an Oxford Nanopore platform. Libraries with different insert sizes were constructed for each platform, and the sequencing reads were subsequently assembled to generate the complete genome. Annotation was performed with Prokka, and gene function was analyzed using the GO and KEGG databases. KL typing of CRKP26 was performed using Kaptive. Comparative mapping of the plasmids was performed using the Basic Local Alignment Tool (BRIG), a visualization tool for genomic comparison. Differential genes were analyzed via BLAST.

### Murine intraperitoneal infection model

All animal procedures were approved by the Institutional Animal Care and Use Committee of Lishui University (protocol number 2025D179).

To evaluate the *in vivo* virulence of the strains, we performed a median lethal dose (LD_50_) assay using BALB/c mice and a modified Karber’s method. Specific pathogen-free male BALB/c mice (6–8 weeks old and weighing 25–30 g) were obtained from the Experimental Animal Center of Lishui University. Briefly, 6–8-week-old male BALB/c mice were randomly assigned to various experimental groups, with six mice per group. The control strain NTUH-K2044, a well-characterized hypervirulent *Klebsiella pneumoniae* isolate originally obtained from a patient with pyogenic liver abscess in Taiwan, and the test strain CRKP26 were cultured, and bacterial suspensions were prepared in saline at an initial concentration of 1 × 10^7^ CFU/mL as the stock solution. This was followed by two-fold serial dilutions to achieve different challenge doses, and the actual inoculum concentration for each dilution was verified by plate counting. Mice were inoculated via intraperitoneal injection with 0.1 mL of the corresponding bacterial suspension, while control group mice received an equal volume of sterile saline. Following inoculation, the mice were monitored continuously for 7 days, with survival status and time of death recorded meticulously. The LD_50_ values for each strain were calculated based on observational data. According to the criteria established by the Chinese expert consensus on the diagnosis, treatment, and prevention of hypervirulent carbapenem-resistant *Klebsiella pneumoniae* infections, an LD_50_ value≤1 × 10^6^ CFU defines a hypervirulent strain.

The mice were housed under standard laboratory conditions with free access to food and water and acclimatized for 7 days before the experiments. Bacterial inocula were prepared by culturing the strains overnight in Luria–Bertani broth, followed by centrifugation and resuspension in sterile normal saline. The concentration was adjusted to 1 × 10^7^ CFU/mL on the basis of the OD_600_ and confirmed by plating. Each mouse was intraperitoneally injected with 100 μL, 200 μL or 300 μL of bacterial suspension. The control mice received normal saline. Groups of 10 mice were used per strain. The mice were monitored at 6-h intervals for 60 h post-injection. Survival was recorded, and Kaplan–Meier survival analysis was performed.

### Neutrophil killing assay

Suspensions of the strains ATCC 13883, a type strain of *Klebsiella pneumoniae* obtained from the American Type Culture Collection (ATCC), NTUH-K2044 and the test isolate strain CRKP26 were adjusted to 0.5 McFarland turbidity and then diluted 1: 100 in phosphate-buffered saline containing 5% serum to approximately 1 × 10^6^ CFU/mL. Suspensions were incubated at 37 °C with shaking at 80 rpm for 20 min prior to use. Human neutrophils were isolated from heparinized whole blood by density gradient centrifugation. Following erythrocyte lysis and washing, the neutrophils were resuspended in PBS containing 10% serum at 1 × 10^6^ cells/mL and used within 1 h. For the killing assay, 300 μL of neutrophil suspension was mixed with 300 μL of bacterial suspension in the experimental group, while 300 μL of PBS was mixed with 300 μL of bacterial suspension in the control group. The samples were incubated at 37 °C with shaking at 180 rpm for 2 h. Following incubation, 0.1% saponin solution was added for neutrophil lysis, the samples were diluted 1: 100, and 10 μL aliquots were plated on blood agar plates. The plates were incubated at 37 °C overnight for colony enumeration.

### Serum bactericidal assay

The serum survival assay was performed as follows. Briefly, bacterial cells from the mid-log phase culture were harvested and adjusted to a concentration of 1.5 × 10^6^ CFU/mL. The bacterial suspension was subsequently mixed with normal human serum (NHS) at a volumetric ratio of 1: 3. The mixture was then incubated at 37 °C for 2 h to allow bactericidal activity. Following incubation, the reaction mixture was serially diluted in sterile phosphate-buffered saline (PBS). To enumerate the viable bacteria, aliquots of the dilutions were plated onto LB agar plates and incubated overnight at 37 °C. The surviving bacteria were quantified by counting the resulting colonies.

### Statistical analysis

All analyses were performed using SPSS 25.0. *p* < 0.05 was considered to indicate statistical significance. Statistically significant differences are indicated as follows: ns, not significant; *p* > 0.05; *: *p* ≤ 0.05; **: *p* ≤ 0.01; ***: *p* ≤ 0.001.

## Results

### NDM-producing *Klebsiella pneumoniae* strains collection and resistance characterization

Between January 2017 and December 2023, 27 NDM-producing *K. pneumoniae* isolates were identified among 94 NDM-producing *Enterobacteriaceae* samples collected from a tertiary hospital in Lishui, China. Carbapenemase gene analysis revealed that 15 isolates (55.6%) carried *bla*_NDM-1_ and 12 (44.4%) harbored *bla*_NDM-5_. Two isolates (7.4%) coproduced both *bla*_KPC-2_ and *bla*_NDM-1_. Additional *β*-lactamase genes detected included *bla*_SHV_ in 25/27 isolates (92.6%), *bla*_TEM_ in 16/27 (59.3%), and *bla*_CTX-M_ in 10/27 (37.0%). The full set of resistance genes detected in each isolate, including carbapenemases, ESBLs, and plasmid-mediated quinolone resistance genes, is provided in Supplementary Table S2.

All 27 isolates were resistant to cephalosporins (ceftazidime, cefotaxime, and cefepime) and carbapenems (meropenem and imipenem). The MICs of carbapenem ranged from 4 to 64 μg/mL for imipenem and from 8 to 256 μg/mL for meropenem (Table 1). The isolates showed variable resistance to aztreonam (15/27, 55.6%), levofloxacin (14/27, 51.9%), and amikacin (3/27, 11.1%) but remained uniformly susceptible to polymyxin B and tigecycline (both 27/27, 100%). The two dual-carbapenemase producers displayed distinct levels of carbapenem resistance: strain CRKP26 exhibited extreme resistance, with meropenem and imipenem MICs of 256 and 64 μg/mL, respectively, which were significantly higher than those of strain CRKP11 (16 and 8 μg/mL, respectively). Strain CRKP26 showed pan-β-lactam resistance, including resistance to aztreonam (MIC ≥256 μg/mL) and amikacin (MIC ≥1,024 μg/mL), and remained susceptible only to colistin (MIC ≤0.5 μg/mL) and tigecycline (MIC = 0.5 μg/mL) (Table 1).

**Table 1:** Summary of the clinical information, molecular characteristics, carbapenemase genes, and antibiotic susceptibility profiles of 27 CRKP isolates.

Strains	Source sample	Patient diagnosis	Isolated year	MLST	Plasmid type	KPC	NDM	CAZ μg/mL	FEP μg/mL	ATM μg/mL	TZP μg/mL	IPM μg/mL	MEM μg/mL	AMK μg/mL	PMB μg/mL	TGC μg/mL	LVX μg/mL
CRKP1	Blood	Ascending Colon Malignancy	2017	ST3216	IncX3	*ND*	NDM-1	≥256	32	64	≥1024/4	32	16	≤4	1	≤0.25	≤1
CRKP2	Blood	Acute Leukemia	2017	ST622	IncX3	*ND*	NDM-5	≥256	128	≤1	≥1024/4	32	16	≤4	≤0.5	≤0.25	≤1
CRKP3	Blood	Cholangiocarcinoma	2017	ST3143	IncX3	*ND*	NDM-1	≥256	16	≤1	512/4	8	16	≤4	1	≤0.25	≤1
CRKP4	Deep Venous Catheter (DVC)	Intestinal Obstruction	2017	ST5116	IncX3	*ND*	NDM-1	≥256	≥256	≤1	≥1024/4	32	32	≤4	1	2	4
CRKP5	Urine	Cardiac Arrest	2017	ST11	IncX3	*ND*	NDM-1	≥256	64	16	≥1024/4	32	32	8	≤0.5	≤0.25	64
CRKP6	Drainage Fluid	Abdominal Pain	2018	ST4	IncX3	*ND*	NDM-1	≥256	128	32	≥1024/4	16	16	≤4	1	≤0.25	≤1
CRKP7	Urine	Abdominal Pain	2018	ST11	IncX3	*ND*	NDM-1	≥256	32	64	64/4	4	8	≤4	≤0.5	≤0.25	8
CRKP8	Blood	Pancreatic Malignancy	2018	ST147	IncX3	*ND*	NDM-5	≥256	≥256	128	≥1024/4	16	64	≤4	≤0.5	≤0.25	32
CRKP9	Urine	Cervical Tumor	2018	ST340	IncX3	*ND*	NDM-5	≥256	≥256	32	≥1024/4	16	32	≤4	≤0.5	2	≥16
CRKP10	Sputum	Pelvic Fracture	2018	ST412	IncX3	*ND*	NDM-1	≥256	≥256	≥256	≥1024/4	8	16	≤4	≤0.5	≤0.25	≤1
CRKP11	Blood	Cerebral Infarction	2019	ST17	X3 FII	KPC-2	NDM-1	≥256	16	128	512/4	8	16	≤4	≤0.5	≤0.25	≤1
CRKP12	Sputum	Malignant Tumor History	2019	ST3924	/	*ND*	NDM-1	≥256	64	≤1	≥1024/4	32	64	≥1,024	1	≤0.25	≤1
CRKP13	Blood	Abdominal Pain	2019	ST3332	IncX3	*ND*	NDM-5	≥256	128	8	≥1024/4	32	128	≥1,024	1	0.5	≥16
CRKP14	Blood	Intracerebral Hemorrhage	2020	ST355	IncX3	*ND*	NDM-1	≥256	128	≤1	≥1024/4	16	32	4	1	0.5	≤1
CRKP15	Sputum	Uremia	2021	ST218	IncX3	*ND*	NDM-1	≥256	32	≤1	≥1024/4	16	32	4	≤0.5	0.5	8
CRKP16	Sputum	Coma	2021	ST5437	IncX3	*ND*	NDM-1	≥256	64	64	≥1024/4	16	32	4	1	1	4
CRKP17	Blood	Cerebral Hernia	2021	ST218	IncX3	*ND*	NDM-1	≥256	32	≤1	512/4	16	32	4	≤0.5	0.5	≤1
CRKP18	Sputum	COPD with Acute Exacerbation	2021	ST485	IncX3	*ND*	NDM-5	≥256	128	16	≥1024/4	32	64	4	≤0.5	0.5	≥16
CRKP19	Intralesional Tissue	Soft Tissue Infection	2021	ST967	IncX3	*ND*	NDM-1	≥256	64	8	≥1024/4	16	32	4	≤0.5	0.5	≤1
CRKP20	Secretion	Acute Respiratory Failure	2021	ST485	IncX3	*ND*	NDM-5	≥256	≥256	32	≥1024/4	32	16	4	1	0.5	≥16
CRKP21	Blood	Basal Ganglia Hemorrhage	2021	ST485	IncX3	*ND*	NDM-5	≥256	≥256	32	≥1024/4	32	64	4	≤0.5	2	≥16
CRKP22	Blood	Pelvic Fracture	2022	ST340	IncX3	*ND*	NDM-5	≥256	16	≤1	512/4	8	16	4	≤0.5	1	≥16
CRKP23	Perianal Secretion	Unspecified Respiratory Failure	2022	ST340	IncX3	*ND*	NDM-5	≥256	16	≤1	256/4	4	8	4	≤0.5	≤0.25	4
CRKP24	Cerebrospinal Fluid (CSF)	Cerebral Hemorrhage History	2022	ST1131	IncX3	*ND*	NDM-5	≥256	≥256	64	512/4	32	16	4	≤0.5	1	≤1
CRKP25	Blood	Cerebral Hemorrhage History	2022	ST1131	IncX3	*ND*	NDM-5	≥256	128	64	512/4	32	16	4	1	1	≤1
CRKP26	Anal Swab	Lung Transplantation Status	2022	ST11	IncX3	KPC-2	NDM-1	≥256	≥256	≥256	≥1024/4	64	256	≥1,024	≤0.5	0.5	8
CRKP27	Blood	Myelodysplastic Syndrome (MDS)	2022	ST5112	IncX3	*ND*	NDM-5	≥256	32	≤1	512/4	32	8	4	≤0.5	0.5	≤1

MLST, multilocus sequence typing; KPC, Klebsiella pneumoniae carbapenemase; NDM, New Delhi metallo-β-lactamase; ND indicates that the specific gene was not detected by PCR or genomic analysis; CAZ, ceftazidime; FEP, cefepime; ATM, aztreonam; TZP, piperacillin/tazobactam; IPM, imipenem; MEM, meropenem; AMK, amikacin; PMB, polymyxin B; TGC, tigecycline; LVX, levofloxacin. Notes: KPC and NDM refer to the presence of the respective carbapenemase-encoding genes; all other abbreviations represent the antibiotics tested in this study.

### MLST, capsular serotyping, and clonal diversity

MLST analysis revealed high clonal diversity, with 19 distinct sequence types (STs) identified among the 27 NDM-producing isolates, in contrast to the predominant clonal expansion of KPC-2-producing ST11 strains observed during national surveillance. The most prevalent STs were ST11, ST340, and ST485, each accounting for 3 isolates (11.1%), followed by ST1131 and ST218, with 2 isolates (7.4%) each. The remaining 14 sequence types were each represented by a single isolate (Table 1). Among the isolates coproducing KPC-2 and NDM-1, CRKP26 belonged to ST11 and was identified as capsular type KL64, whereas CRKP11 belonged to ST17 and capsular type KL25.

### Plasmid types and resistance gene context

To assess plasmid transferability, conjugation and transformation experiments were conducted as previously described (Huang et al., 2020). NDM-carrying plasmids from 26/27 isolates (96.3%) were successfully transferred to recipient strains, indicating high horizontal transfer potential. Plasmid replicon typing revealed that IncX3 was the predominant type in 25/27 isolates (92.6%), with one isolate (CRKP11) harboring an IncX3/IncFII hybrid replicon (Table 1). The remaining isolate failed to yield a transferable plasmid in conjugation assays and therefore could not be assigned a specific replicon type.

Analysis of the *bla*_NDM_ genetic context revealed a conserved core structure (*bla*_NDM_-*bla*_MBL_-*trpF*) with variable flanking regions. Ten distinct genetic contexts were identified: seven variants in *bla*_NDM-1_-carrying isolates and three in *bla*_NDM-5_-carrying isolates. With respect to CRKP26, one of the two dual-carbapenemase producers, *bla*_KPC-2_ was located on an IncFII/IncR plasmid within a *NTE*_KPC_-I chimeric transposon structure (Huang et al., 2020), and *bla*_NDM-1_ was located on an IncX3 plasmid with the typical *ISAba125*-*bla*_NDM-1_-*bla*_MBL_-*trpF*-*dsbC* genetic context.

### Chromosomal and plasmid architecture in CRKP26

We performed whole-genome sequencing on CRKP26. The genome comprised a 5,529,569-bp chromosome with 57.31% GC content and 5,292 predicted ORFs, along with six plasmids ranging from 5.6 to 198.7 kb in length. Chromosomal analysis revealed virulence genes, including genes encoding siderophores (*iroN*, *iutA*, *iron-enterobactin*, and *yersiniabactin*), and a type VI secretion system (T6SS). The chromosome also harbored the *β*-lactamase gene *bla*_SHV-11_ and a glycine-aspartate (GD) mutation at positions 137–138 in the *OmpK36* porin gene, a mutation previously associated with reduced carbapenem permeability (Wong et al., 2019). The whole-genome sequencing data for strain CRKP26 have been deposited at the National Microbiology Data Center (NMDC) under accession number NMDC60216001.

**Figure 1:**
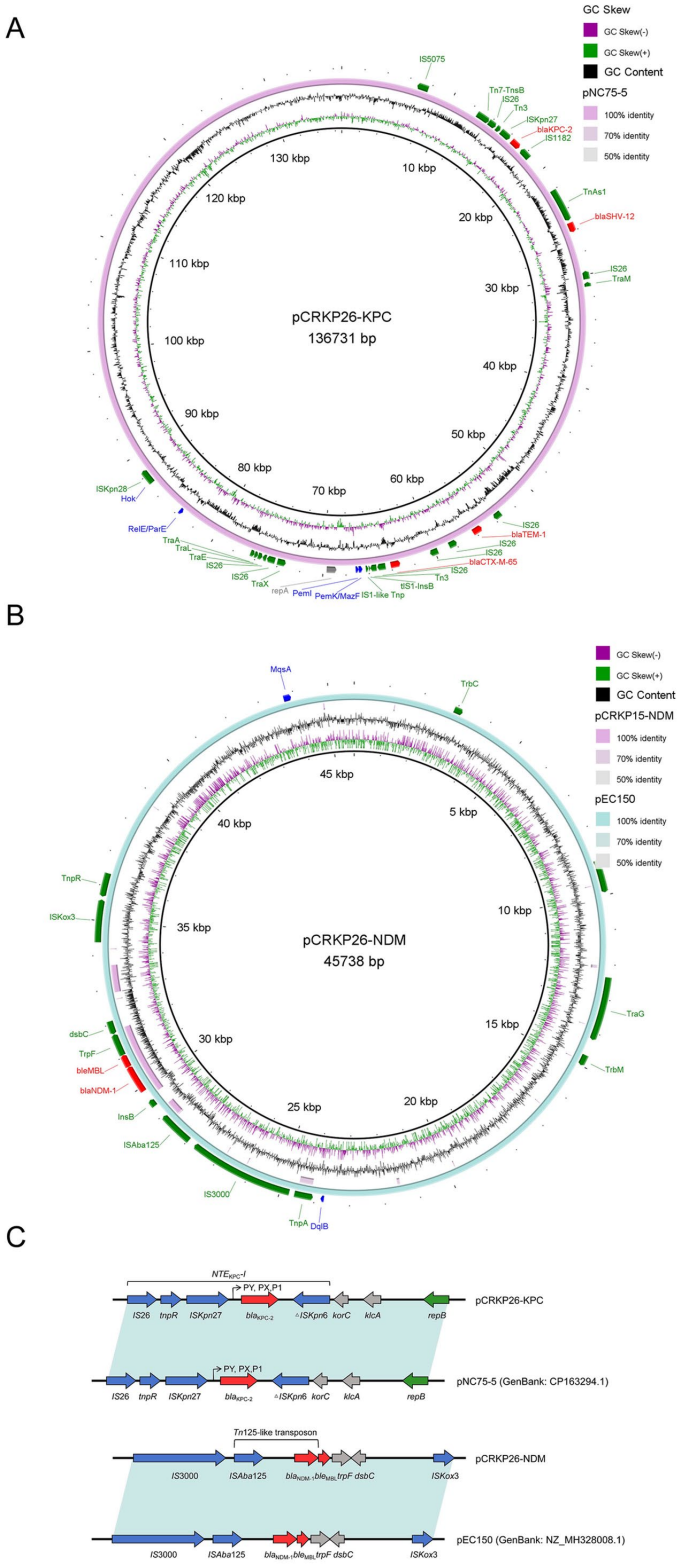
Carbapenemase-encoding plasmid structures in CRKP26. Circular maps of carbapenemase-encoding plasmids. **(A)** IncFII/IncR hybrid plasmid carrying *bla*_KPC-2_ (136731 bp). **(B)** IncX3 plasmid carrying *bla*_NDM-1_ (45738 bp). Mobile genetic elements (green), resistance genes (red), virulence genes (blue), and replication origin (gray) are indicated. **(C)** Linear genomic comparison of the *bla*_KPC-2_ and *bla*_NDM-1_ genetic environments.

Whole-genome sequencing identified six plasmids with distinct sizes, incompatibility groups, and genetic features, which are summarized in Table 2. pCRKP26-KPC was a IncFII/IncR hybrid plasmid. Bioinformatics analysis revealed that this plasmid harbored *bla*_KPC-2_, *bla*_SHV-66_, *rmtB*, *bla*_TEM-1b_, and *bla*_CTX-M-65_, conferring resistance to carbapenems, extended-spectrum cephalosporins, and aminoglycosides (Figure 1A). pCRKP26-NDM was a IncX3 plasmid carrying *bla*_NDM-1_ and *ble*_MBL_, which confer resistance to β-lactams, including carbapenems (Figure 1B). pCRKP26-vir is a IncHI1B plasmid encoding the hypervirulence determinants *iucABCD* and *iutA*. pCRKP26-3 carried the quinolone resistance gene *qnrS1* and the β-lactamase gene *bla*_TEM-215_. However, no major resistance or virulence genes were detected in pCRKP26-5 or pCRKP26-6, which contained only replication and mobilization genes.

**Table 2:** Genomic characteristics of the chromosome and plasmids identified in strain CRKP26.

Replicon	Name	Size (bp)	Incompatibility group	GC content (%)	Resistance genes	Virulence genes
Chromosome	CRKP26-chromosome	5,529,569	Chromosome	57.31	*bla*_SHV-11_ *, katG, alr/ddl, fosA5, strA, tet34, bacA*	*iroN, iutA, enterobactin, yersiniabactin, T6SS*
Plasmid 1	pCRKP26-vir	198,670	IncHI1B	50.19	*ND*	*iucABCD, iutA, hemB*
Plasmid 2	pCRKP26-KPC	136,731	IncFII/IncR	53.36	*bla*_KPC-2_, *bla*_SHV-66_, *rmtB*, *bla*_TEM-1b_, *bla*_CTX-M-65_	*ibeB*
Plasmid 3	pCRKP26-3	84,876	IncFII	54.12	*qnrS1*, *bla*_TEM-215_, *dfrA14, sul2, tetR, tetG*	*ND*
Plasmid 4	pCRKP26-NDM	45,738	IncX3	46.60	*bla*_NDM-1_, *ble*_MBL_	*ND*
Plasmid 5	pCRKP26-5	11,970	ColRNAI	55.59	*ND*	*ND*
Plasmid 6	pCRKP26-6	5,596	Non-typeable	51.14	*ND*	*ND*

ND indicates that the specific gene was not detected by PCR or genomic analysis.

Comparative sequence analysis revealed that pCRKP26-KPC shared 100% nucleotide identity (100% coverage) with pNC75-5 (GenBank: CP163294.1) from a *K. pneumoniae* strain isolated in Jiangxi Province (Figure 1A), suggesting interprovincial dissemination. In contrast, pCRKP26-NDM was identical (100% identity, 100% coverage) to pEC150 (GenBank: NZ_MH328008.1), an NDM-1 plasmid previously identified in *Enterobacter cloacae* from the same intensive care unit in 2015 (Figure 1B), indicating interspecies horizontal transfer within the healthcare facility.

For clarity, the local genetic environments of *bla*_KPC-2_ and *bla*_NDM-1_ are further illustrated as a linear comparison (Figure 1C) (Luo et al., 2024).

### Virulence plasmid comparison and gene content

**Figure 2:**
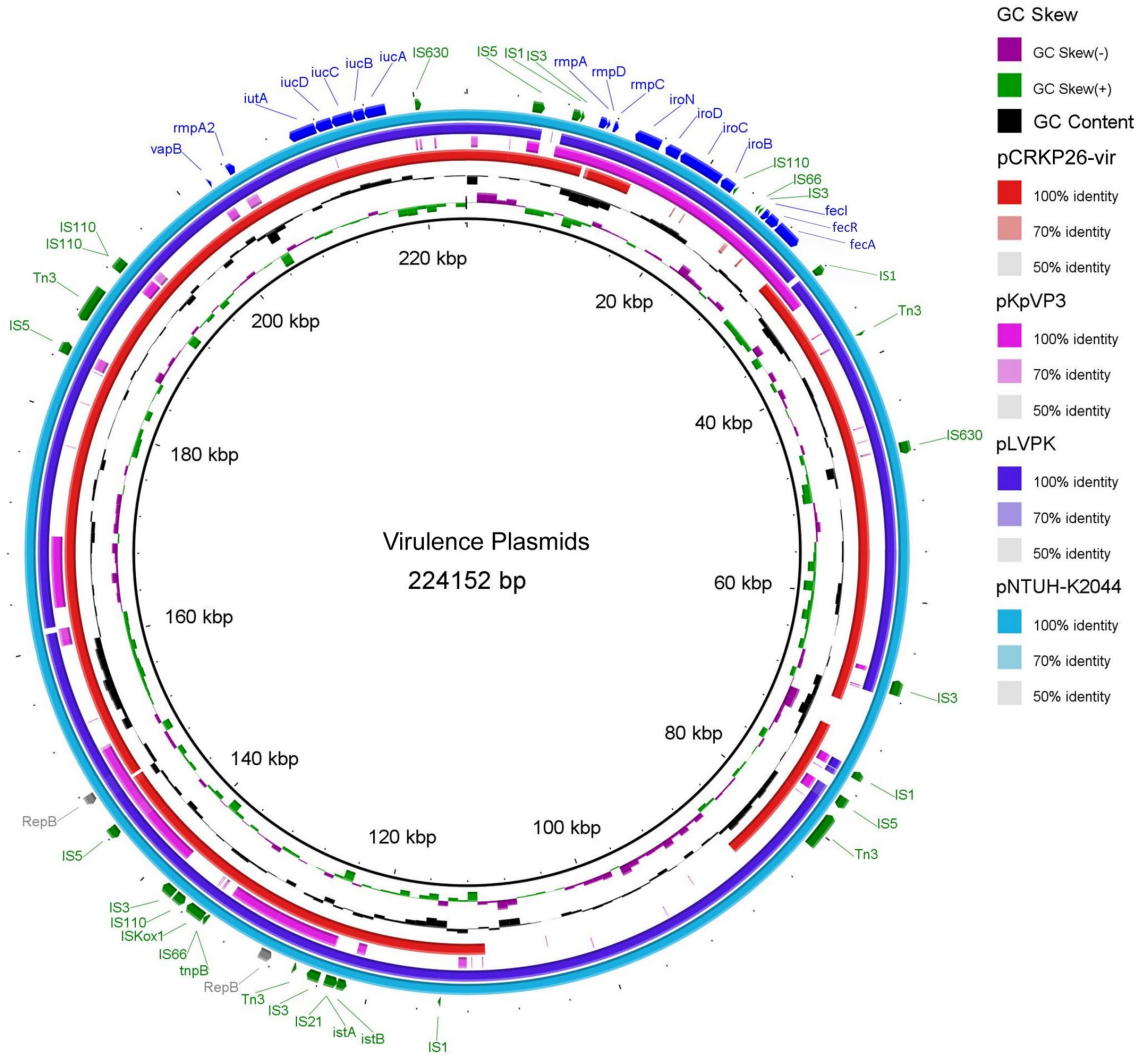
Comparative genomic analysis of the virulence plasmid pCRKP26 with three prototypical hypervirulence plasmids, pNTUH-K2044 (KpVP-1), pLVPK (KpVP-2) and KpVP-3. The circular map depicts the architecture of pCRKP26-vir (red ring) compared with that of the prototypical plasmids pLVPK (dark blue ring), pNTUH-K2044 (light blue ring), and KpVP-3 (purple ring). The colored regions indicate sequence homology. Blank sectors represent deletions in pCRKP26, including the *iro, fec, sil, pco,* and *cus* clusters, while the aerobactin system (*iuc-iut*) and capsule regulators (*rmp*) are retained. Mobile genetic elements (green), resistance genes (red), virulence genes (blue), and replication origin (gray) are indicated.

The CRKP26 virulence plasmid was compared with three reference virulence plasmids: the classical pNTUH-K2044 (KpVP-1), the prototypical pLVPK virulence plasmid (KpVP-2), and the KpVP-3-type virulence plasmid (CP 132963) (Figure 2).

The CRKP26 virulence plasmid (pCRKP26-vir, 198,670 bp), belonging to the IncHI1B incompatibility group, retained the aerobactin biosynthesis and transport system (*iucABCD-iutA-def*) and capsule regulatory genes (*rmpA*, *rmpC*, and *rmpD*). The retained *rmpA* gene shared 84.89% nucleotide identity with the reference gene in pNTUH-K2044 (97% coverage).

pCRKP26-vir retained the core aerobactin system (*iucABCD-iutA*) and capsule regulators (*rmpA*) but lacked the accessory salmochelin (*iroB/C/D/N*) and ferric citrate uptake systems (*fecI/R/A*). This deletion pattern suggests that primary iron acquisition mechanisms are preserved while secondary iron uptake pathways are lost.

### Virulence assessment of CRKP26

**Figure 3:**
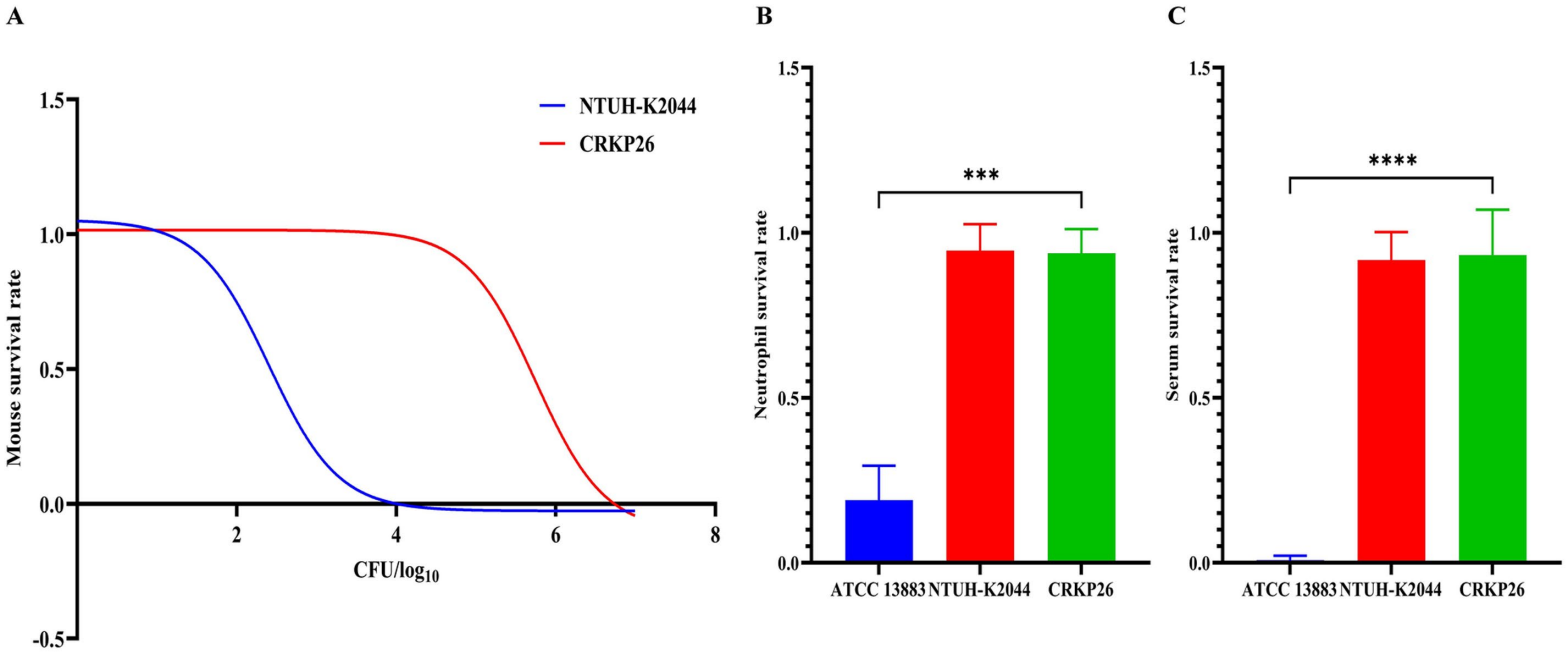
Virulence characterization of CRKP26 using murine infection models and immune evasion assays. **(A)** BALB/c mice (*n* = 6 per group) were intraperitoneally challenged with serial dilutions of the test strain CRKP26 or the hypervirulent strain NTUH-K2044. The median lethal dose (LD_50_) was calculated after a 7-day observation period. **(B)** Neutrophil killing assay showing bacterial survival rates after 2 h of incubation with human neutrophils. **(C)** Serum bactericidal assay showing resistance to complement-mediated killing after 2 h of incubation in normal human serum. NS, normal saline control. Statistical significance was determined by the one-way ANOVA for *in vitro* assays (**B,C**, three independent experiments). **, *p* < 0.01; ***, *p* < 0.001; ****, *p* < 0.0001.

The *in vivo* virulence of the strains was assessed by determining the median lethal dose (LD_50_) in a murine model. The reference hypervirulent strain NTUH-K2044 exhibited high virulence with an LD_50_ of 2.53 × 10^2^ CFU. Consistent with the definition of hypervirulence (LD_50_ ≤ 1 × 10^6^ CFU) outlined by the Chinese expert consensus, the test strain CRKP26 also demonstrated a hypervirulent phenotype, with a calculated LD_50_ of 5.55 × 10^5^ CFU. Although the LD_50_ value of CRKP26 was approximately 3-log_10_ higher than that of NTUH-K2044, indicating a moderately attenuated lethality, it unequivocally qualifies as a hypervirulent strain (Figure 3A).

The virulence of CRKP26 was evaluated using mice intraperitoneally infected with bacteria at three concentrations over a 60-h observation period (Supplementary Figures S1–S3). At 1 × 10^6^ CFU, NTUH-K2044 caused rapid mortality, with a survival rate of only 20% by 48 h; however, CRKP26 showed attenuated virulence, resulting in a 90% survival rate, and the survival rate after ATCC 13883 infection was 100% through 60 h (Supplementary Figure S1). At 2 × 10^6^ CFU, NTUH-K2044 resulted in 100% mortality by 48 h, whereas CRKP26 demonstrated intermediate virulence, with 40% survival at 60 h, which was significantly greater than that of ATCC 13883, which resulted in a 100% survival rate (*p* < 0.01; Supplementary Figure S2). At the highest concentration (3 × 10^6^ CFU), both NTUH-K2044 and CRKP26 caused 100% mortality within 24 h, demonstrating comparably high virulence. In contrast, 90% survival was observed through 60 h after ATCC 13883 infection, with CRKP26 infection resulting in a significantly lower survival rate (*p* < 0.0001; Supplementary Figure S3).

In a neutrophil killing assay, the survival rate of ATCC 13883 was 19.1%, while the survival rates of NTUH-K2044 and CRKP26 were significantly greater (91.9 and 90.7%, respectively). Compared with ATCC 13883, CRKP26 was significantly more resistant to neutrophil-mediated death (*p* < 0.001; Figure 3B). In the serum bactericidal assay, the survival rate of ATCC 13883 was less than 1%, whereas the survival rates of NTUH-K2044 and CRKP26 were high (91.7 and 93.2%, respectively). Serum resistance was significantly greater for CRKP26 than for ATCC 13883 (*p* < 0.0001; Figure 3C). Both *in vivo* and *in vitro* virulence assessments demonstrated that the virulence of CRKP26 was significantly greater than that of ATCC 13883 but slightly lower than that of the hypervirulent strain NTUH-K2044.

## Discussion

Carbapenem-resistant hypervirulent *Klebsiella pneumoniae* (CR-hvKP) poses a critical clinical threat because of the convergence of extensive drug resistance and enhanced pathogenicity, particularly within the globally disseminated ST11 clone (Gu et al., 2018). In this study, we characterized a hypervirulent ST11 *K. pneumoniae* strain (CRKP26) isolated from a lung transplant patient. This strain simultaneously harbors a combination of three critical factors: dual carbapenemase production (*bla*_KPC-2_ and *bla*_NDM-1_), an *OmpK36* GD mutation, and plasmid-borne hypervirulence determinants. This convergence corresponded with extreme carbapenem resistance and a hypervirulent phenotype, which was demonstrated in in vitro and *in vivo* models. To our knowledge, this represents the first report of an *OmpK36* GD mutation within a hypervirulent *K. pneumoniae* strain coproducing KPC and NDM, highlighting a concerning development in CR-hvKP evolution.

CRKP26 exhibited extensive resistance to carbapenems, ceftazidime-avibactam, and aztreonam, leaving only polymyxin B and tigecycline as therapeutic options. This resistance pattern severely limits treatment options for carbapenem-resistant infections. The extensive resistance observed for CRKP26 can be attributed to the coproduction of the carbapenemases KPC-2 and NDM-1, which provide complementary resistance mechanisms. KPC-2 is susceptible to avibactam, but the activity of NDM-1, a metallo-*β*-lactamase, is not inhibited by avibactam, conferring resistance to ceftazidime-avibactam. Similarly, aztreonam remains stable in the presence of NDM-1 but is hydrolyzed by KPC-2 (Li et al., 2024; Zhang et al., 2025). This complementary pattern effectively eliminates all β-lactam-based therapeutic options, which is consistent with previous reports on KPC/NDM-coproducing strains. Additionally, CRKP26 harbors an *OmpK36* GD mutation, which has been associated with enhanced carbapenem resistance and reduced susceptibility to newer β-lactam/β-lactamase inhibitor combinations such as meropenem-vaborbactam (Wong et al., 2019; Rogers et al., 2023; Wong et al., 2022; Sun et al., 2017). Compared with CRKP11, another KPC-2/NDM-1 coproducing isolate in our collection, CRKP26 exhibited higher carbapenem MICs. Both strains coproduce the same carbapenemases, but only CRKP26 harbors the *OmpK36* GD mutation. This suggests that porin alteration may further amplify resistance. The combination of dual carbapenemase production and porin mutation results in a virtually untreatable resistance profile, highlighting the critical need for novel therapeutic strategies and stringent infection control measures.

Beyond extensive drug resistance, CRKP26 exhibited a hypervirulent phenotype in infection models. In the murine intraperitoneal infection model, apparent pathogenicity was observed. Although strain CRKP26 demonstrated a moderately lower lethality compared to the classic hypervirulent strain NTUH-K2044, it still meets the criteria for a hypervirulent strain as defined by the Chinese expert consensus on the diagnosis, treatment, and prevention of hypervirulent carbapenem-resistant *Klebsiella pneumoniae* infections, which specifies an LD_50_ value≤1 × 10^6^ CFU. This hypervirulent nature was confirmed *in vitro*, where CRKP26 showed robust resistance to both neutrophil-mediated and serum-mediated killing at levels comparable to those of NTUH-K2044. This slight attenuation observed *in vivo* compared with NTUH-K2044 may be attributed to genetic variations in its virulence determinants. The hypervirulence plasmid in CRKP26 lacked the salmochelin iron acquisition system (*iroB/C/D/N*) present in pLVPK, which has been shown to contribute to enhanced virulence (Abbas et al., 2024; Huang et al., 2023). Additionally, although CRKP26 harbors the *rmpA* gene, this gene shares only 84.89% nucleotide identity with *rmpA* in NTUH-K2044. This low identity indicates significant mutations in the *rmpA* gene. Similarly, CRKP26 tested negative in the string test. Studies have shown that *rmpA* mutations drive the transition from hypermucoviscous CR-hvKp to low-mucoviscous CR-hvKp, a pattern observed in most clinical CR-hvKp strains in China (Liu et al., 2022; Song et al., 2024). The absence of salmochelin combined with *rmpA* mutations may explain the reduced virulence and loss of hypermucoviscosity in CRKP26 compared with NTUH-K2044.

CRKP26 belongs to ST11 and the KL64 capsular serotype (ST11-KL64), which is associated with the most prevalent clone of carbapenem-resistant hypervirulent *K. pneumoniae* in China (Liao et al., 2020). Recent genomic analysis of this lineage further highlights its evolutionary plasticity, with some isolates accumulating a more complex array of multiple resistance genes alongside hypervirulence determinants (Luo et al., 2024). ST11 strains exhibit high transmissibility and the ability to acquire both resistance and virulence determinants through horizontal gene transfer (Zhou et al., 2020; Fu et al., 2019), posing a major public health threat. Epidemiological investigations of KPC/NDM coproducing strains have shown that ST11 accounts for more than 60% of such isolates (Li et al., 2024; Zhang et al., 2025). Concurrently, the acquisition of pLVPK-like virulence plasmids can readily convert ST11 carbapenem-resistant strains into hypervirulent strains (Zhou et al., 2020; Gu et al., 2018). The identification of CRKP26 demonstrates the capacity of this high-risk ST11 clone to accumulate hypervirulence factors and dual-carbapenemase resistance (KPC-2 and NDM-1) and maintain its high potential for dissemination.

In summary, our data reveal the emergence of a hypervirulent ST11-KL64 *K. pneumoniae* strain (CRKP26) with a virtually untreatable resistance profile. This phenotype is driven by a novel convergence of resistance and virulence factors. The strain simultaneously harbors plasmid-mediated dual carbapenemases (KPC-2 and NDM-1), a chromosomal *OmpK36* GD mutation, and plasmid-borne hypervirulence determinants. The continued evolution of such multidrug-resistant and virulent pathogens within a high-risk ST11 clone poses a significant public health threat. These findings highlight the critical need for surveillance strategies to include both chromosomal porin mutations and resistance/virulence plasmids to mitigate the spread of these formidable pathogens.

## Data Availability

The datasets presented in this study can be found in online repositories. The names of the repository/repositories and accession number(s) can be found at: https://www.nmdc.cn/, NMDC60216001.
