# Supplementary material for: First identification of an ST11-KL64 hypervirulent Klebsiella pneumoniae strain coproducing KPC-2 and NDM-1 with an OmpK36 GD mutation
Source: Front Microbiol. 2026 Feb 16;17:1755521. doi: 10.3389/fmicb.2026.1755521 (PMC12950690; doi:10.3389/fmicb.2026.1755521)
Supplement: Supplementary file 1 [file Data_Sheet_1.docx]

**Supplementary Material**


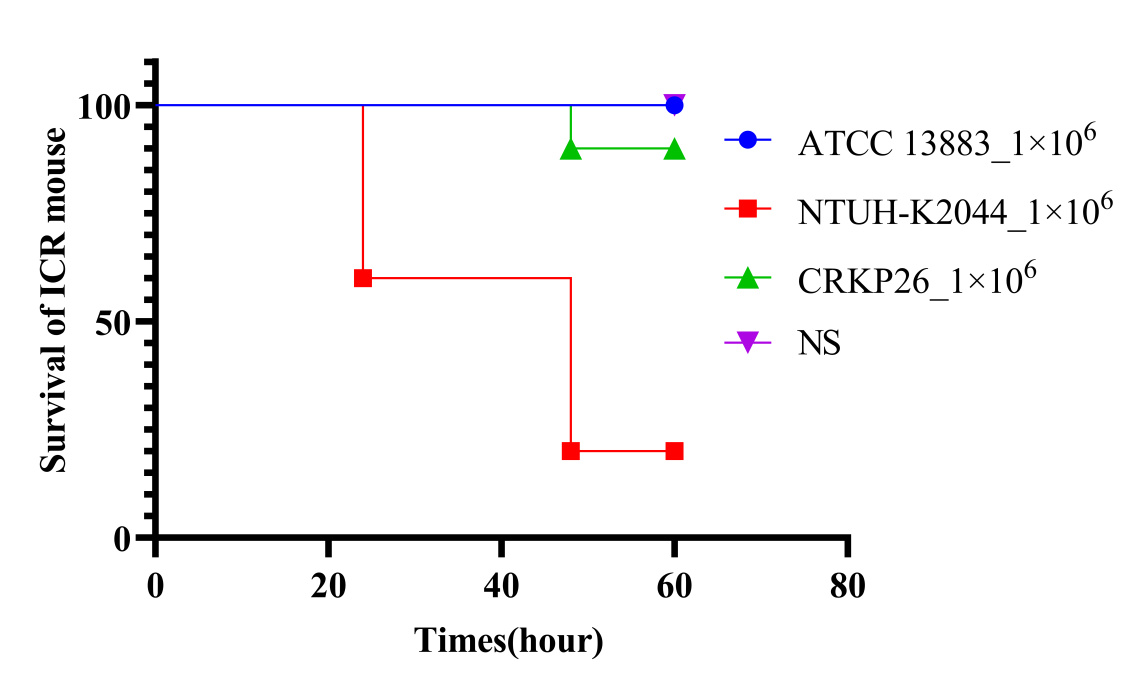


**Figure S1.** Kaplan‒Meier survival curves of BALB/c mice following intraperitoneal challenge with bacterial inocula of 1×10^6^ CFU. Statistical significance was determined by the log-rank (Mantel‒Cox) test for survival curves (n = 10 mice per group).


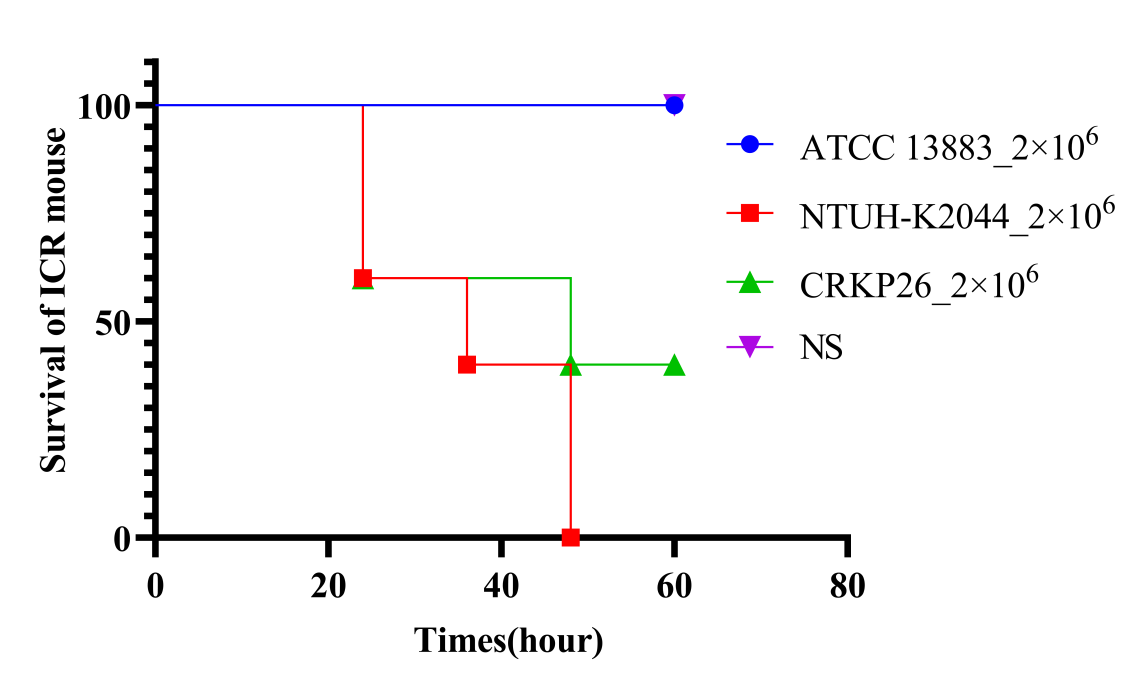


**Figure S2.** Kaplan‒Meier survival curves of BALB/c mice following intraperitoneal challenge with 2×10^6^ CFU of bacterial inocula. Statistical significance was determined by the log-rank (Mantel‒Cox) test for survival curves (n = 10 mice per group).

**
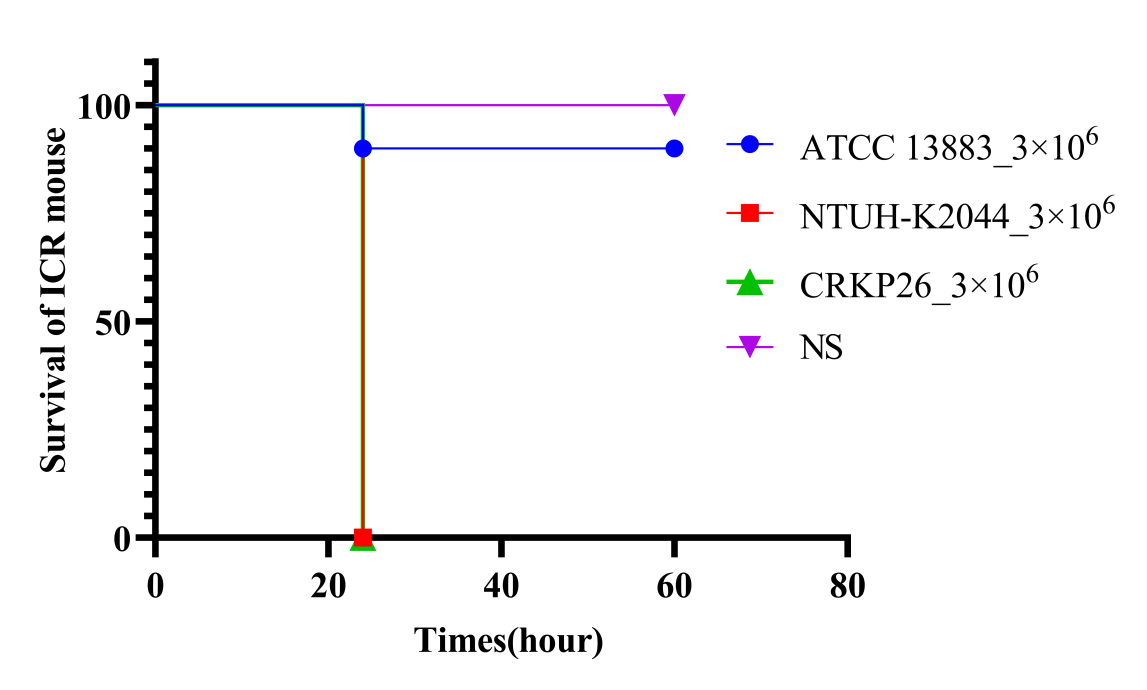
**

**Figure S3.** Kaplan‒Meier survival curves of BALB/c mice following intraperitoneal challenge with 3×10^6^ CFU of bacterial inocula. Statistical significance was determined by the log-rank (Mantel‒Cox) test for survival curves (n = 10 mice per group).

**Table S1.** PCR Primers for Resistance Genes

| Gene | Primer | Primer sequence | Size |
| --- | --- | --- | --- |
| *bla*_KPC_ | *KPC*-F | 5’-GCTACACCTAGCTCCACCTTC-3’ | 989 bp |
|  | *KPC*-R | 5’-ACAGTGGTTGGTAATCCATGC-3’ |  |
| *bla*_NDM_ | *NDM*-F | 5’-GGCTTTTGAAACTGTCGCAC-3’ | 1056 bp |
|  | *NDM*-R | 5’-TTCCAACTCGTGGCAAAGCC-3’ |  |
| *bla*_IMP_ | *IMP*-F | 5’-CTACCGCAGCAGAGTCTTTG-3’ | 587 bp |
|  | *IMP*-R | 5’-AACCAGTTTTGCCTTACCAT-3’ |  |
| *bla*_VIM_ | *VIM*-F | 5’-AGTGGTGAGTATCCGACAG-3’ | 261 bp |
|  | *VIM*-R | 5’-ATGAAAGTGCGTGGAGAC-3’ |  |
| *bla*_GES_ | *GES*-F | 5’-GGTGCAGCTTAGCGACAATG-3’ | 467 bp |
|  | *GES*-R | 5’-GCGTAATCTCTCTCCTGGGC-3’ |  |
| *bla*_SME_ | *SME*-F | 5’-ACTTTGATGGGAGGATTGGC-3’ | 551 bp |
|  | *SME*-R | 5’-ACGAATTCGAGATCACCAG-3’ |  |
| *bla*_IMI_ | *IMI*-F | 5’-CCATTCACCCATCACAAC-3’ | 440 bp |
|  | *IMI*-R | 5’-CTACCGCATAATCATTTGC-3’ |  |
| *bla*_SIM_ | *SIM*-F | 5’-TACAAGGGATTCGGCATCG-3’ | 570 bp |
|  | *SIM*-R | 5’-TAATGGCCTGTTCCCATGTG-3’ |  |
| *bla*_GIM_ | *GIM*-F | 5’-TCGACACACCTTGGTCTGAA-3’ | 477 bp |
|  | *GIM*-R | 5’-AACTTCCAACTTTGCCATGC-3’ |  |
| *bla*_SPM_ | *SPM*-F | 5’-CTGCTTGGATTCATGGGCGC-3’ | 783 bp |
|  | *SPM*-R | 5’-CCTTTTCCGCGACCTTGATC-3’ |  |
| *bla*_OXA-23_ | *OXA-23*-F | 5’-ACTTGCTATGTGGTTGCTTCTCTT-3’ | 797 bp |
|  | *OXA-23*-R | 5’-TTCAGCTGTTTTAATGATTTCATCA-3’ |  |
| *bla*_OXA-24_ | *OXA-24*-F | 5’-CGATCAGAATGTTCAAGCGC-3’ | 559 bp |
|  | *OXA-24*-R | 5’-ACGATTCTCCCCTCTGCGC-3’ |  |
| *bla*_OXA-58_ | *OXA-58*-F | 5’-CGATCAGAATGTTCAAGCGC-3’ | 529 bp |
|  | *OXA-58*-R | 5’-ACGATTCTCCCCTCTGCGC-3’ |  |
| *bla*_CTX-M_ | *CTX-M*-F | 5’-TTTGCGATGTGCAGTACCAGTAA-3’ | 554 bp |
|  | *CTX-M*-R | 5’-CGATATCGTTGGTGGTGCCATA-3’ |  |
| *bla*_CTX-M-1_ | *CTX-M-1*-F | 5’-AAAAATCACTGCGTCAGTTCAC-3’ | 867 bp |
|  | *CTX-M-1*-R | 5’-ACAAACCGTTGGTGACGATT-3’ |  |
| *bla*_CTX-M-2_ | *CTX-M-2*-F | 5’-ATGATGACTCAGAGCATTCG-3’ | 857 bp |
|  | *CTX-M-2*-R | 5’-TCAGAAACCGTGGGTTACGA-3’ |  |
| *bla*_CTX-M-9_ | *CTX-M-9*-F | 5’-TATTGGGAGTTTGAGATGGT-3’ | 933 bp |
|  | *CTX-M-9*-R | 5’-TCCTTCAACTCAGCAAAAGT-3’ |  |
| *bla*_SHV_ | *SHV*-F | 5’-AGCCGCTTGAGCAAATTAAAC-3’ | 713 bp |
|  | *SHV*-R | 5’-ATCCCGCAGATAAATCACCAC-3’ |  |
| *bla*_TEM_ | *TEM*-F | 5’-CATTTCCGTGTCGCCCTTATTC-3’ | 800 bp |
|  | *TEM*-R | 5’-CGTTCATCCATAGTTGCCTGAC-3’ |  |
| *qnr*A | *qnr*A-F | 5’-TCGGCAAAGGTCAGGTCACAGC-3’ | 479 bp |
|  | *qnr*A-R | 5’-GCAAGAGGATTTCTCACGCCAGGAT-3’ |  |
| *qnr*B | *qnr*B-F | 5’-GATCGTGAAAGCCAGAAAGG-3’ | 469 bp |
|  | *qnr*B-R | 5’-ACGATGCCTGGTAGTTGTCC-3’ |  |
| *qnr*C | *qnr*C-F | 5’-GAATTATTCCCATAAAACG-3’ | 509 bp |
|  | *qnr*C-R | 5’-GCTCCCAAAAGTCATC-3’ |  |
| *qnr*D | *qnr*D-F | 5’-CGAGATCAATTTACGGGGAATA-3’ | 582 bp |
|  | *qnr*D-R | 5’-AACAAGCTGAAGCGCCTG-3’ |  |
| *qnr*S | *qnr*S-F | 5’-ACGACATTCGTCAACTGCAA-3’ | 417 bp |
|  | *qnr*S-R | 5’-TAAATTGGCACCCTGTAGGC-3’ |  |
| *qep*A | *qep*A-F | 5’-CGTGTTGCTGGAGTTCTTC-3’ | 402 bp |
|  | *qep*A-R | 5’-CTGCAGGTACTGCGTCATG-3’ |  |
| *aac(6')-Ib-cr* | *aac(6')-Ib-cr*-F | 5’-TTGCGATGCTCTATGAGTGG-3’ | 478 bp |
|  | *aac(6')-Ib-cr*-R | 5’-CTCGAATGCCTGGCGTGTTT-3’ |  |

**Table S2.** Resistance Genes in 27 *bla*_NDM_-positive Isolates

| Stains | *bla*NDM | *bla*KPC | *bla*VIM | *bla*IMP | *bla*GES | *bla*SME | *bla*IMI | *bla*SIM | *bla*GIM | *bla*SPM | *bla*OXA-23 | *bla*OXA-24 | *bla*OXA-58 | *bla*CTX-M | *bla*CTX-M-1 | *bla*CTX-M-2 | *bla*CTX-M-9 | *bla*TEM | *bla*SHV | *qnr*A | *qnr*B | *qnr*C | *qnr*D | *qnr*S | *qep*A | *aac(6')-Ib-cr* |
| --- | --- | --- | --- | --- | --- | --- | --- | --- | --- | --- | --- | --- | --- | --- | --- | --- | --- | --- | --- | --- | --- | --- | --- | --- | --- | --- |
| CRKP1 | + | - | - | - | - | - | - | - | - | - | - | - | - | - | - | - | - | - | + | - | - | - | - | + | - | - |
| CRKP2 | + | - | - | - | - | - | - | - | - | - | - | - | - | - | - | - | - | - | - | - | - | - | - | - | - | - |
| CRKP3 | + | - | - | - | - | - | - | - | - | - | - | - | - | - | - | - | - | - | + | - | - | - | - | - | - | - |
| CRKP4 | + | - | - | - | - | - | - | - | - | - | - | - | - | - | - | - | - | - | + | - | - | - | - | + | - | - |
| CRKP5 | + | - | - | - | - | - | - | - | - | - | - | - | - | - | - | - | - | - | + | - | - | - | - | - | - | + |
| CRKP6 | + | - | - | - | - | - | - | - | - | - | - | - | - | + | + | - | - | + | - | - | - | - | - | + | - | - |
| CRKP7 | + | - | - | - | - | - | - | - | - | - | - | - | - | - | - | - | - | - | + | - | - | - | - | - | - | + |
| CRKP8 | + | - | - | - | - | - | - | - | - | - | - | - | - | + | + | - | - | + | + | - | + | - | - | + | - | + |
| CRKP9 | + | - | - | - | - | - | - | - | - | - | - | - | - | - | - | - | - | + | + | - | - | - | - | - | - | + |
| CRKP10 | + | - | - | - | - | - | - | - | - | - | - | - | - | + | + | - | - | + | + | - | - | - | - | + | - | - |
| CRKP11 | + | + | - | - | - | - | - | - | - | - | - | - | - | - | - | - | - | - | + | - | - | - | - | + | - | - |
| CRKP12 | + | - | - | - | - | - | - | - | - | - | - | - | - | - | - | - | - | + | + | - | - | - | - | - | - | - |
| CRKP13 | + | - | - | - | - | - | - | - | - | - | - | - | - | - | - | - | - | - | + | - | - | - | - | - | - | + |
| CRKP14 | + | - | - | - | - | - | - | - | - | - | - | - | - | - | - | - | - | - | + | - | - | - | - | - | - | - |
| CRKP15 | + | - | - | - | - | - | - | - | - | - | - | - | - | - | - | - | - | - | + | - | + | - | - | + | - | + |
| CRKP16 | + | - | - | - | - | - | - | - | - | - | - | - | - | + | + | - | - | + | + | - | + | - | - | + | - | + |
| CRKP17 | + | - | - | - | - | - | - | - | - | - | - | - | - | - | - | - | - | - | + | - | - | - | - | + | - | - |
| CRKP18 | + | - | - | - | - | - | - | - | - | - | - | - | - | + | + | - | - | + | + | - | + | - | - | + | - | + |
| CRKP19 | + | - | - | - | - | - | - | - | - | - | - | - | - | + | + | - | - | + | + | - | - | - | - | + | - | + |
| CRKP20 | + | - | - | - | - | - | - | - | - | - | - | - | - | + | + | - | - | + | + | - | + | - | - | + | - | + |
| CRKP21 | + | - | - | - | - | - | - | - | - | - | - | - | - | + | + | - | - | + | + | - | + | - | - | + | - | + |
| CRKP22 | + | - | - | - | - | - | - | - | - | - | - | - | - | - | - | - | - | + | + | - | - | - | - | - | - | + |
| CRKP23 | + | - | - | - | - | - | - | - | - | - | - | - | - | - | - | - | - | + | + | - | - | - | - | - | - | + |
| CRKP24 | + | - | - | - | - | - | - | - | - | - | - | - | - | + | + | - | - | + | + | - | - | - | - | - | - | - |
| CRKP25 | + | - | - | - | - | - | - | - | - | - | - | - | - | + | + | - | - | + | + | - | - | - | - | - | - | - |
| CRKP26 | + | + | - | - | - | - | - | - | - | - | - | - | - | - | - | - | - | + | + | - | - | - | - | + | - | - |
| CRKP27 | + | - | - | - | - | - | - | - | - | - | - | - | - | - | - | - | - | + | + | - | - | - | - | + | - | + |

Abbreviations: The following resistance genes were screened in this study: carbapenemase genes (*bla*_NDM_, *bla*_KPC_, *bla*_VIM_, *bla*_IMP_, *bla*_GES_, *bla*_SME_, *bla*_IMI_, *bla*_SIM_, *bla*_GIM_, *bla*_SPM_, *bla*_OXA-23_, *bla*_OXA-24_, and *bla*_OXA-58_); extended-spectrum β-lactamase (ESBL) genes (*bla*_CTX-M_, *bla*_CTX-M-1_, *bla*_CTX-M-2_, *bla*_CTX-M-9_, *bla*_TEM_, and *bla*_SHV_); plasmid-mediated quinolone resistance (PMQR) genes (*qnrA, qnrB, qnrC, qnrD, qnrS, qepA*); and the aminoglycoside resistance gene *aac(6')-Ib-cr*.
